# Possible Role of Non-Muscle Alpha-Actinins in Muscle Cell Mechanosensitivity

**DOI:** 10.1371/journal.pone.0096395

**Published:** 2014-04-29

**Authors:** Irina V. Ogneva, Nikolay S. Biryukov, Toomas A. Leinsoo, Irina M. Larina

**Affiliations:** Department of Molecular and Cell Biomedicine, State Scientific Center of Russian Federation Institute of Biomedical Problems of the Russian Academy of Sciences, Moscow, Russia; University of Vienna, Max F. Perutz Laboratories, Austria

## Abstract

The main hypothesis suggested that changes in the external mechanical load would lead to different deformations of the submembranous cytoskeleton and, as a result, dissociation of different proteins from its structure (induced by increased/decreased mechanical stress). The study subjects were fibers of the soleus muscle and cardiomyocytes of Wistar rats. Changes in external mechanical conditions were reconstructed by means of antiorthostatic suspension of the animals by their tails for 6, 12, 18, 24 and 72 hours. Transversal stiffness was measured by atomic force microscopy imaging; beta-, gamma-actin, alpha-actinin 1 and alpha-actinin 4 levels in membranous and cytoplasmic fractions were quantified by Western blot analysis; expression rates of the corresponding genes were studied using RT-PCR. Results: In 6 hours, alpha-actinin 1 and alpha-actinin 4 levels decreased in the membranous fraction of proteins of cardiomyocytes and soleus muscle fibers, respectively, but increased in the cytoplasmic fraction of the abovementioned cells. After 6–12 hours of suspension, the expression rates of beta-, gamma-actin, alpha-actinin 1 and alpha-actinin 4 were elevated in the soleus muscle fibers, but the alpha-actinin 1 expression rate returned to the reference level in 72 hours. After 18–24 hours, the expression rates of beta-actin and alpha-actinin 4 increased in cardiomyocytes, while the alpha-actinin 1 expression rate decreased in soleus muscle fibers. After 12 hours, the beta- and gamma-actin content dropped in the membranous fraction and increased in the cytoplasmic protein fractions from both cardiomyocytes and soleus muscle fibers. The stiffness of both cell types decreased after the same period of time. Further, during the unloading period the concentration of nonmuscle actin and different isoforms of alpha-actinins increased in the membranous fraction from cardiomyocytes. At the same time, the concentration of the abovementioned proteins decreased in the soleus muscle fibers.

## Introduction

Exposure to zero gravity may have a negative impact on different organs and tissues in humans and other species (for instance, in rodents). In rodents, antiorthostatic suspension is accompanied by similar effects on a number of systems (for example, on muscle, bone and partially the cardiovascular system) [1].

Skeletal muscles (as a specialized organ maintaining posture and providing motor function) are particularly prone to the negative effects of zero gravity. Exposure of soleus muscle to conditions of microgravity for long periods of time has been shown to result in significant weight loss and atrophic changes [2], [3], [4]. Moreover, a decrease in functional capacity has been reported for the whole muscle [5], [6] and for its isolated fibers [7]. The adverse changes developing in the soleus muscle are mainly due to disturbances in its electrical activity. However, one should note that adverse changes may develop not only in the soleus muscle (the electrical activity of which is seriously affected under conditions of antiorthostatic suspension) [8], but in the tibialis anterior muscle (despite the fact that electrical activity of the latter increases under the same conditions), as well as in the medial gastrocnemius muscle (the electrical activity of which doesn't change during suspension) [8]. We have previously shown that changes to the structure of the fibers of calf muscles were correlated with disturbances in their electrical activity [9]. However, the structure of the submembranous cytoskeleton (which was assessed using an integral mechanical parameter–its transverse stiffness) was damaged in all of the abovementioned muscles [9]. Such observations may be related to external mechanical stress reduction on calf muscles under conditions of antiorthostatic suspension.

Exposure to microgravity results in different disturbances in the cardiovascular system in humans, mainly a fluid shift in the cranial direction [10], [11] and changes of systolic output [12], [13], [14]. Clinical manifestations of such effects are not so apparent in rodents in the model of antiorthostatic suspension, but even under such experimental conditions numerous investigators reported a volume overload on the heart [15], [16], [17]. We demonstrated in our previous studies that transverse stiffness of the contractile apparatus of rat left ventricle cardiomyocytes increased after 72 hours of antiorthostatic suspension (moreover, the stiffness of the submembranous cytoskeleton occurred much earlier–after 24 hours of suspension) [18]. We suppose that such changes may be related to volume overload on the heart (external mechanical stress due to tension), which may take place, at least, at early stages of antiorthostatic suspension.

Summarizing the experimental data, one can suggest that changes in the structure of the contractile apparatus of both skeletal muscle fibers and cardiomyocytes are mainly related to the functional activity of these cells. However, changes of cortical cytoskeleton structure (which appear much earlier than changes of the contractile apparatus) may be linked to the levels of external mechanical stress on these cells.

It should be noted that the structure of the submembranous cytoskeleton of muscle cells (either fibers of skeletal muscles or cardiomyocytes) is generally similar to the structure of the cortical cytoskeleton of non-muscle cells, except for several particular sites (in projection of M- and Z-line membrane). Actin (beta- and gamma-) is the major protein of the cortical cytoskeleton, which forms stress fibrils that bind to each other through different actin-binding proteins. Published data state that changes in external mechanical conditions may result in a reorganization of the cortical cytoskeleton [19], [20], [21], [22], [23], [24], [25].

However, mechanisms of interactions between cells and the external mechanical field have not been fully studied yet, and the answer to the following question is still unclear: what is the fundamental difference between cell responses to external mechanical stress increases/decreases? Therefore, the main hypothesis of our study was that changes in the external mechanical load would lead to different deformations of the submembranous cytoskeleton and, as a result, dissociation of different proteins from its structure (induced by increased/decreased mechanical stress). Concurrently, an *in vivo* model of antiorthostatic suspension of rodents allows simulating the increase/decrease of external mechanical stress in the same organism, in the cells possessing the same structure (cardiomyocytes and fibers of skeletal muscles, in particular fibers of the soleus muscle).

## Materials and Methods

The experiments have been performed with the tissue of the left ventricle and soleus muscle of a Wistar rat (n = 42 animals) weighing 225 to 255 g. To simulate the microgravity conditions in rodents, antiorthostatic suspension was used according to the Ilyin-Novikov method modified by Morey-Holton et al. [1]. Control animals were housed at the vivarium conditions and received standard food and water ad libitum. The duration of suspension was 6, 12, 18, 24 and 72 hours. The following groups were created: «Control», «6h», «12h», «18h», «24h» and «72h», with seven animals in each group. There were no changes in mass of the left ventricle during the whole period of antiorthostatic suspension. The soleus muscle mass was remained unchanged up to the 24 hours of the suspension, after 3 days of suspension, soleus mass decreased by 25.4%.

All procedures with animals were approved by the biomedical ethics committee of the State Research Center of Russia Institute of Biomedical Problems of the Russian Academy of Sciences.

### Serum Corticosterone Level by Enzymeimmunoassay Analysis

Blood was collected in EDTA-containing tubes after rat decapitation. Serum was collected by centrifugation at 3000×g for 10 min and stored at −80°C prior to analysis. Total corticosterone levels in serum samples from rats of control and all suspension groups were determined using a commercially available enzymeimmunoassay kit (IDS GmbH, Germany) according to the manufacturer’s instructions. Corticosterone levels were assayed in duplicate.

### Transversal Stiffness by Atomic Force Microscopy

Cardiomyocytes were obtained from a part of the tissue of the rat’s left ventricle using a standard method [26], [27], without using Triton X-100. Soleus muscle was extracted from tendon to tendon and treated according with a standard method described by Stevens et al. [28].

Before the experiments, samples were stored at −20°C in a buffer containing equal parts of: relaxation solution R (20 mM MOPS, 170 mM of potassium propionate, 2.5 mM of magnesium acetate, 5 mM of K_2_EGTA, 2.5 mM of ATP) and glycerol.

On the day of the experiment, the samples were transferred to solution R where single glycerynized soleus fibers and cardiomyocytes were singled out.

In order to measure the transversal stiffness, the obtained soleus fibers and cardiomyocytes were fixed on the bottom of the liquid cell of the atomic force microscope, attaching their tips with special Fluka shellac wax-free glue (Sigma, Germany).

Measurements of transversal stiffness were conducted using the Solver-P47-Pro platform (NT-MDT, Russia) in the contact mode with the indentation depth of 150 nm [29]. We tested at least 17 cells from each sample (n = 119 at least from each group).

The results were processed in a special program in MatLab 6.5.

### Protein Content by Western Blotting

In order to determine the protein content, partions of the rat’s left ventricle and soleus muscle were frozen at the temperature of liquid nitrogen. The method described in Vitorino et al. [30] was used to prepare tissue extracts and obtain the cytoplasmic and membrane fraction of proteins. After it we measured total protein concentration in the each sample by using spectrophotometry. Denaturing polyacrylamide gel electrophoresis was performed using the Laemmli method and the Bio-Rad system (USA). Basing on the measured concentration of the total fraction protein content, equal amounts of protein were added to each hole. We estimated changes of relative content of each protein in comparison with the respective one in the group “Control”. For the standardization, bands, which were correlate with different experimental groups, on the membrane were compared with the band of the group “Control” on this membrane. There were seven compositions for quantifying results. Transfer to nitrocellulose membranes was performed using the method of Towbin et al. [31].

In order to quantify each protein, specific monoclonal primary antibodies based on mouse immunoglobulins were used (Santa Cruz Biotechnology, Inc.) in the manufacturer-recommended dilutions: 1∶300 for beta-actin, 1∶100 for gamma-actin, 1∶100 for alpha-actinin-1, and 1∶100 for alpha-actinin-4. For secondary antibodies, we used biotinylated goat antibodies against mice IgG (Santa Cruz Biotechnology, Inc.) diluted 1∶5000.

Afterwards, all membranes were treated with streptavidin conjugated with horseradish peroxidase (Sigma, Germany) diluted 1∶5000. Protein bands were identified using 3,3′-diaminobenzidine (Merck, USA). ImageJ software were used for quantifying western-blots.

### Expression Level by Real-time PCR

For estimating the expression level, total cellular RNA from rat frozen tissues of soleus muscle and heart left ventricles was isolated using an RNeasy Micro Kit (Qiagen, Germany) according to the manufacturer’s protocol. Amplifications of transcripts were performed using 500 ng of total RNA and one-step reverse transcription RT-PCR system (Qiagen, Germany) according to the manufacturer’s protocol. The PCR primer sequences used in this study were designed using Primer3Plus software (Table 1). Melt curves were performed to insure fidelity of the PCR product. The 2(-Delta Delta C(T)) method [32] was used to determine fold difference. We added this information to the manuscript.

**Table 1:** RT-PCR primers and products.

Gene	Direction	Primer sequence (5′…3′)	Product size, bp
Actn1	Forward	ggtcagcagcaacctcctc	167
	Reverse	tctttctccaccttctctcca	
Actn4	Forward	accctgaacagactcccttg	168
	Reverse	gatcgacaagcctccatctc	
Actb	Forward	gctgcgttttacaccctttc	218
	Reverse	gtttgctccaaccaactgct	
Actg	Forward	ctggtggatctctgtgagca	184
	Reverse	tcaggagggaagaaaccaga	

### Statistical Analysis

The results obtained during the experiments were statistically processed with ANOVA, using a post-hoc t-test with a confidence level p<0.05 to evaluate the certainty of difference between the groups. The data was represented as M±SE, where M is the average arithmetic value and SE is the average value error.

## Results

### Rat Stress Levels (Table 2) under Short-term Antiorthostatic Suspension

To determine rat stress levels exposed to the short-term antiorthostatic suspension, we determined the serum corticosterone levels. The results (Table 2) indicate that there was a slight increase in corticosterone level by 31% (p<0.1) after 6 hours of antiorthostatic suspension. After 12 hours of unloading, corticosterone levels returned to values of the group «Control» and then during suspension did not differ from the control levels.

**Table 2:** Rat serum corticosterone levels (ng/ml) after short-term antiorthostatic suspension.

Control (n = 7)	6 h (n = 7)	12 h (n = 7)	18 h (n = 7)	24 h (n = 7)	72 h (n = 7)
42±7	55±4*	41±4	38±4	40±4	42±5

*p<0.1 as compared to the group «Control».

### Dynamics of the Transversal Stiffness of Rat Left Ventricle Cardiomyocytes and Soleus Muscle Fibers under Short-term Antiorthostatic Suspension (Table 3, Fig. 1)

**Figure 1:**
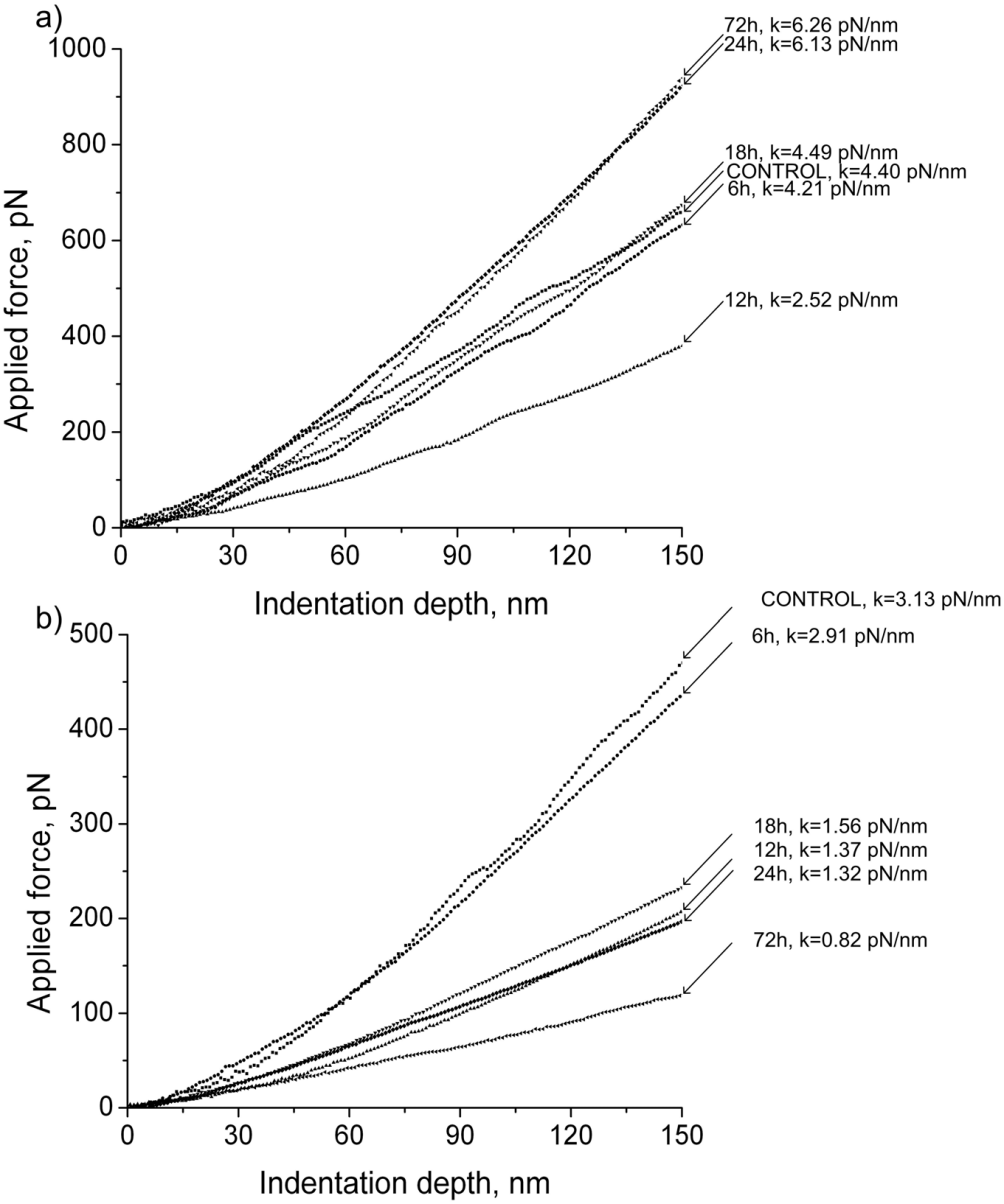
Typical force curves obtained under transversal stiffness measurements of rat left ventricle cardiomyocytes (A) and soleus fibers (B) of the rats under short-term antiorthostatic suspension. (A) – changes in the transversal stiffness of cardiomyocytes were sharp and in different directions: first, the stiffness was reduced and then restored to control levels and further exceeded it. (B) – the transversal stiffness of soleus muscle fibers was reduced in steps: falling in 12 hours and remaining at this level up to 24 hours of suspension and then dropping back to 72 hours of gravitational unloading.

Transversal stiffness of rat left ventricle cardiomyocytes (Table 3, Fig. 1A) after 6 hours of suspension did not differ from the control group level. After 12 hours stiffness decreased by 42% (p<0.05), and after 18 hours it significantly increased in comparison with the group «12h» and reached the group «Control». In addition, during suspension cardiomyocyte stiffness continued to grow, exceeding the control level after 24 hours to 41% (p<0.05) and after 72 hours to 44% (p<0.05).

**Table 3:** Transversal stiffness (pN/nm) rat’s left ventricle cardiomyocytes and soleus muscle fibers under short-term antiorthostatic suspension.

Tissue Group	Cardiomyocytes	Soleus fibers
Control (n = 7)	4.39±0.14	3.12±0.09
6 h (n = 7)	4.24±0.18	2.93±0.11
12 h (n = 7)	2.53±0.08*↓	1.38±0.09*
18 h (n = 7)	4.51±0.19&	1.57±0.12*
24 h (n = 7)	6.17±0.17* ^/&^	1.31±0.09*
72 h (n = 7)	6.31±0.13* ^/&^	0.81±0.04*

*p<0.05 as compared to the group «Control»,

& p<0.05 as compared to the group «12 h».

Transversal stiffness of the rat soleus muscle fibers after 6 hours of antiorthostatic suspension did not differ from the control level. After 12 hours of gravitational disuse, stiffness (Table 3, Fig. 1B) decreased by 56% (p<0.05) in comparison with group «Control» and continued to decline, falling in 72 hours by 74% (p<0.05) as compared to the control group level and by 41% (p<0.05) in comparison with group «12h».

Thus, cardiomyocyte and soleus fiber transversal stiffness decreased after 12 hours of suspension, but subsequently cardiomyocyte stiffness increased and exceeded control levels; soleus fiber stiffness further decreased.

### Cytoskeletal Protein Content Dynamic of Left Ventricle Cardiomyocytes and Soleus Muscle Fibers of Rats after Short-term Gravitational Unloading

**Figure 2:**
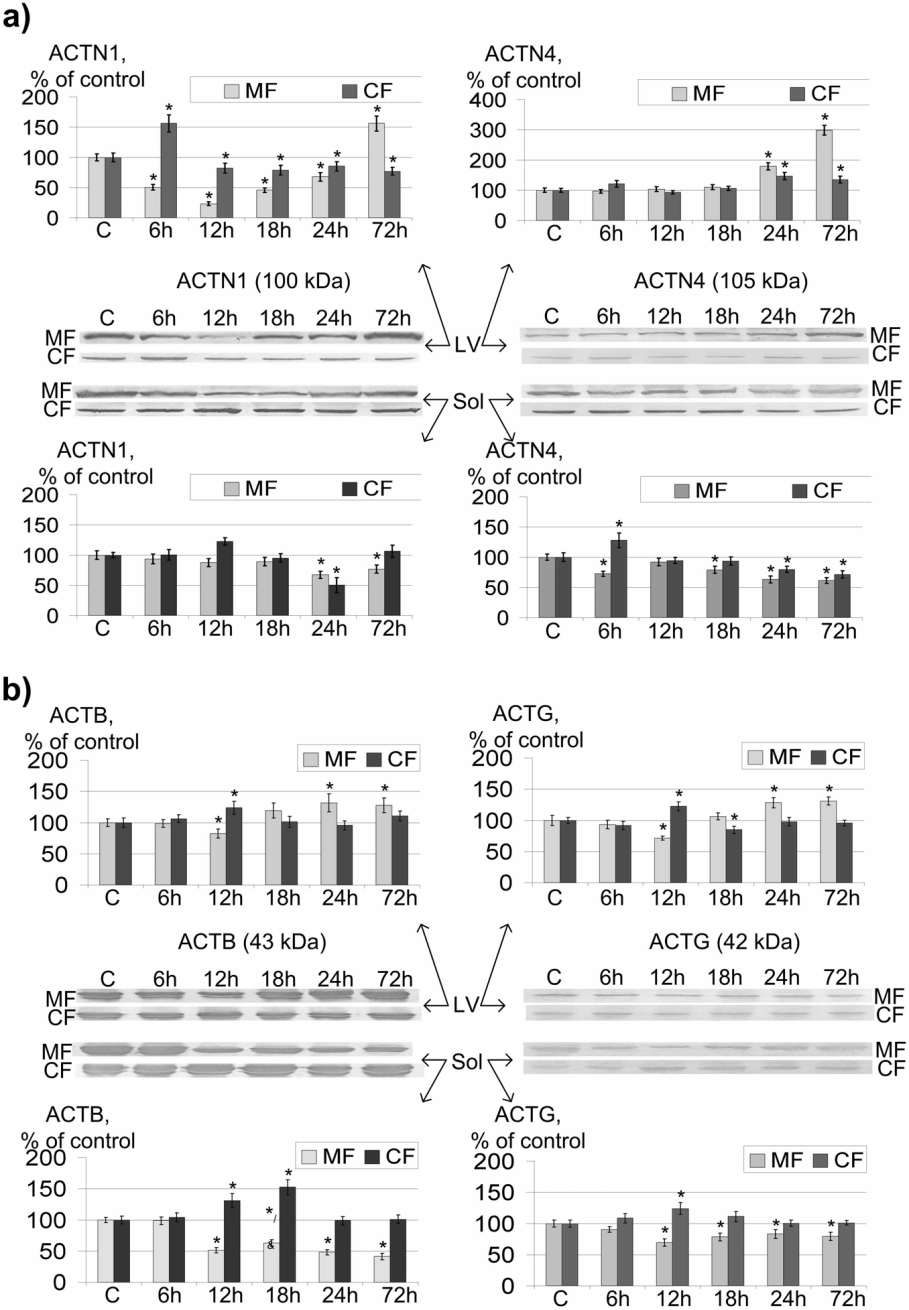
Relative protein content in the membrane protein fraction (MF) and cytoplasmic protein fraction (CF) of left ventricle cardiomyocytes (LV) and soleus muscle fibers (Sol) of rats after short-term gravitational unloading and typical Western blot pictures. * – p<0.05 as compared to the group «Control» – indicated as «C» in this figure, ^&^ – p<0.1 as compared to the group «12h». A – alpha-actinin-1 (ACTN1) and alpha-actinin-4 (ACTN4) content, B – beta-actin (ACTB) and gamma-actin (ACTG) content.

The alpha-actinin-1 (ACTN1) content (Fig. 2A) in the membrane fractions of rat left ventricles were reduced by 49.6% (p<0.05) after 6 hours of antiorthostatic suspension. At the same time, the content of this protein in the cytoplasmic fraction increased by 56.4% (p<0.05) compared to control levels. It continued to decrease in the membrane fraction (down to 23.1% (p<0.05) relative to control levels), but it also decreased in the cytoplasmic fraction [down to 82.3% (p<0.05) in comparison with control] after 12 hours of suspension. It subsequently continued to grow in the membrane fraction and exceeded control levels by 56% (p<0.05) after 72 hours of unloading; however, ACTN1 levels were reduced by 22.9% in the cytoplasmic fraction (p<0.05).

The ACTN1 content change in the membrane fraction of soleus fibers was detected only after 24 hours of antiorthostatic suspension: it had decreased by 32.7% (p<0.05) from control levels in the membrane fraction and by 49.7% in the cytoplasmic fraction. However, after 72 hours of unloading the ACTN1 content in the cytoplasmic fraction returned to control levels and remained low in the membrane fraction [by 23.1% (p<0.05)].

The left ventricle alpha-actinin-4 (ACTN4) content was constant for 24 hours of gravitational unloading, then it increased by 79% (p<0.05) in the cytoplasm and by 46.7% (p<0.05) in membrane fractions. After 72 hours, it exceeded control levels by 198.8% (p<0.05) in the membrane fraction and by 35.3% (p<0.05) in the cytoplasmic fraction.

At the same time, the ACTN4 content in soleus fibers changed after 6 hours of suspension. It was reduced by 27.4% (p<0.05) in the membrane fraction and increased by 28.2% (p<0.05) in the cytoplasmic fraction. The protein content in both the cytoplasmic and membrane fractions returned to control levels after 12 hours of suspension. After that, the protein content began to reduce. It reached its minimum after 72 hours of suspension and was reduced by 38.7% (p<0.05) in the membrane fraction and by 28.7% (p<0.05) in the cytoplasmic fraction in comparison with control levels.

The relative beta-actin (ACTB) content (Fig. 2B) in rat left ventricle membrane protein fractions decreased significantly by 17.2% (p<0.05) after 12 hours of suspension. It increased at the same time in cytoplasmic fractions by 23.8% (p<0.05). The ACTB content in membrane fractions were restored to control levels after 18 hours, and exceeded it after 24 and 72 hours of suspension. The ACTB content in cytoplasmic fractions also restored to control levels after 18 hours and remained constant throughout all the following periods.

The relative ACTB content in the membrane fraction of soleus fibers decreased by 48.6% (p<0.05) and increased by 31.3% (p<0.05) in the cytoplasmic fraction after 12 hours of antiorthostatic suspension. In the membrane fraction, it increased by 11.7% (p<0.1) compared to 12-hour-data, but it continued to decrease to 72 hours and subsequently became 41.3% relative to control levels, being consistently equal to control values in the cytoplasmic fraction.

The gamma-actin (ACTG) content in both fractions of both cardiomyocytes and soleus muscle fibers demonstrated similar dynamics as the ACTB relative content.

### Dynamic of Expression Level of Genes Encoding Cytoskeletal Proteins in Left Ventricle Cardiomyocytes and Soleus Muscle Fibers of Rats after Short-term Gravitational Unloading

**Figure 3:**
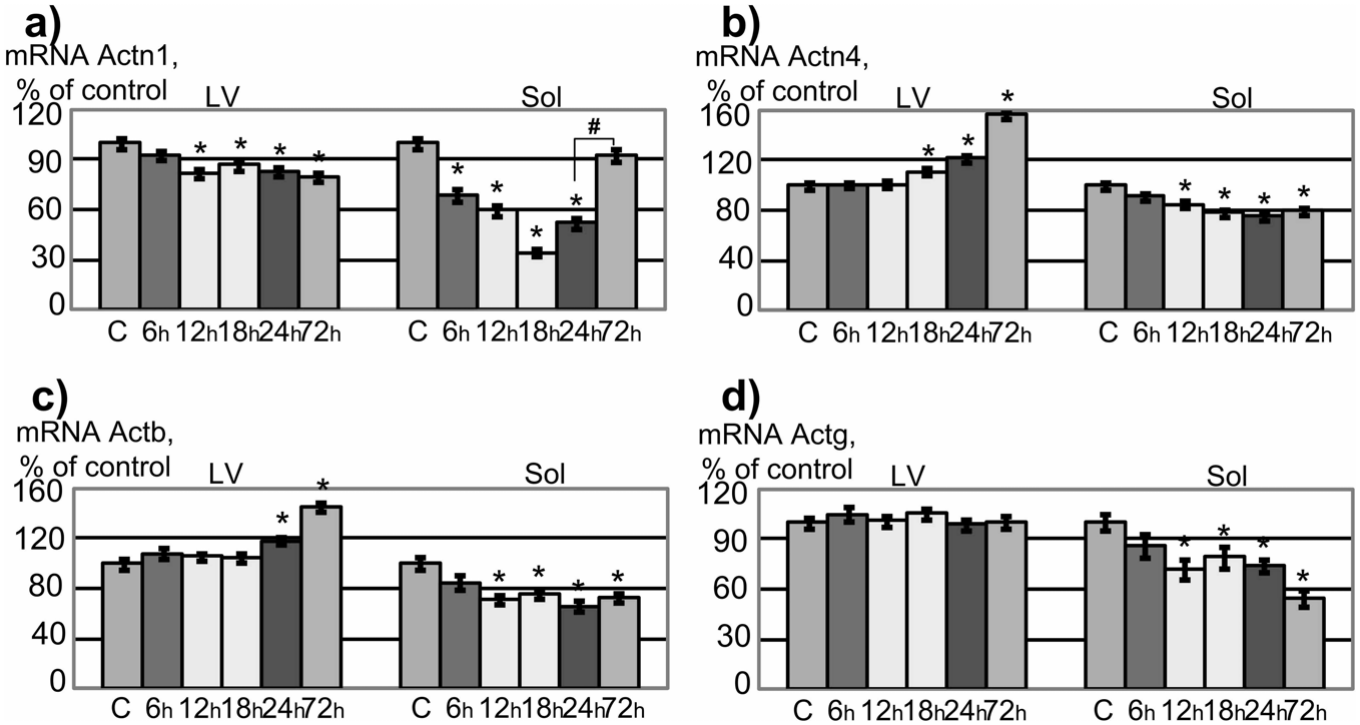
Expression level of genes encoding cytoskeletal proteins in left ventricle cardiomyocytes (LV) and soleus muscle fibers (Sol) of rats after short-term gravitational unloading. *–p<0.05 as compared to the group «Control» – indicated as «C» in this figure, ^#^–p<0.05 as compared to the group «24h». A – alpha-actinin-1 gene (Actn1), B – alpha-actinin-4 gene (Actn4), C – beta-actin gene (Actb), D – gamma-actin gene (Actg).

The expression level of the alpha-actinin-1 gene (actn1) (Fig. 3A) in rat left ventricles decreased by 20% (p<0.05) after 12 hours of antiorthostatic suspension and remained at this level up to 72 hours of the disuse. At the same time, Actn1 mRNA levels in soleus fibers decreased by 30% (p<0.05) after 6 hours and continued to decrease up to 18 hours. After that, it began to grow and after 72 hours there was no change in comparison with group «Control» and significantly increased compared to group «24h».

The expression level of the alpha-actinin-4 gene (actn4) (Fig. 3B) in rat left ventricles increased compared to group «Control» after 18 hours of hindlimb suspension and continued to grow, exceeding control levels after 72 hours by 57% (p<0.05). In contrast, in soleus fibers Actn4 mRNA levels decreased by 22% (p<0.05) in comparison with the group «Control» after 12 hours of the suspension and remained unchanged during follow-up disuse.

The expression level of the beta-actin gene (actb) (Fig. 3C) in cardiomyocytes increased by 18% (p<0.05) after 24 hours and by 45% (p<0.05) after 72 hours of the hindlimb unloading compared to control levels. In the soleus fibers, Actb mRNA content decreased by 29% (p<0.05) after 12 hours of suspension in comparison with the group «Control» and remained at this level up to 72 hours of the disuse.

The expression level of the gamma-actin gene (actg) (Fig. 3D) in cardiomyocytes remained unchanged throughout the period of disuse. In the soleus muscle fiber, the content of Actg and Actb mRNA showed similar changes: it decreased after 12 hours by 30% (p<0.05) and remained at this level up to 72 hours of the disuse.

## Discussion

The problem of mechanosensitivity is still one of the most under investigated aspects in the field of cell biology. Issues concerning gravity changes are very complicated, as the intensity of its effect is directly proportional to the value of cell mass and, subsequently, very low. The situation when gravity values do not change, but its direction does, seems to be even more ambiguous. Although in cells organized in tissues, such changes of the gravity vector direction initiate a number of other processes related to changes of neural activation (for cells of the soleus muscle) or redistribution of hydrostatic pressure (for cardiomyocytes). Subsequently, identification of a cell mechanosensor is an extremely difficult objective.

In particular, extracellular matrix and membrane proteins, components of ion channels, cytoskeletal compounds and intracellular structures may serve as mechanosensors [33], [34], [35], [36], [37], [38], [39], [40], [41], [42], [43]. But almost all possible mechanisms of primary mechanotransduction depend on the condition of the submembranous cortical cytoskeleton, the structural integrity of which determines the mechanical properties of the certain type of cell and is reflected in the cell stiffness.

### Short-term Antiorthostatic Suspension does not Cause Stress in Rats

As this study was conducted in animals under a short period of external impact, issues on the stress level may seem absolutely natural. That is why we determined the serum corticosterone levels in rats. The obtained data suggest that almost no stress may result from this type of impact. This fact fully complies with data available in the literature, as obtained in studies with more prolonged periods of impact [44]. Some insignificant corticosterone level increase, noted after 6 hours of antiorthostatic suspension, is not attributed to an animal’s stay in an atypical posture, but to the contact with an investigator.

### Transverse Stiffness of the Cortical Cytoskeleton of Muscle Cells and Content of Non-muscle Actin Isoforms

Stiffness of both cell types significantly decreased after 12 hours of antiorthostatic suspension. After 18 hours of suspension, the transverse stiffness of cardiomyocytes returned to control levels. The stiffness of soleus muscle fibers slightly enhanced (but insignificantly in comparison to the value reported at 12 hours of suspension). Further, during the unloading period the cardiomyocyte stiffness was elevated in comparison to the control group. On the contrary, stiffness of the soleus muscle fibers decreased.

Changes in stiffness of the cortical cytoskeleton itself, probably, are of no fundamental functional importance for muscle cells due to its insignificant contribution to mechanical features of this type of cell (which are mostly determined by their contractile apparatus). However, changes in the cortical cytoskeleton stiffness may initiate a number of signaling pathways, particularly regulate ion channels activity. By means of patch clamp techniques, it was shown that actin microfilaments participated in the regulation of chloride ion channels [45], [46], Na+/K+-ATPase [47], voltage-gated sodium channels in brain cells [48], and in the sodium channels of the cells of the reabsorption epithelium [49]. Fragmentation of actin filaments (associated with plasmatic membrane) induced by a cytoplasmic actin-binding Ca2+-sensitive protein (similar to endogenous gelsolin) may constitute the main factor, enhancing the activity of sodium channels in response to an increase of intracellular calcium ion concentrations in the K562 cell line [50], [51].

According to the data by Collinsworth et al. [52], the transversal stiffness of muscle fiber’s sarcolemma can decrease due to the destruction of sub-membrane actin cytoskeleton. That is why we tested non-muscle actin content.

The decrease in non-muscle actin isoform content in the membranous fraction of cardiomyocytes after 12 hours of suspension was associated with its increasing in the cytoplasmic fraction. A reverse process took place after 18 hours of suspension. One should note that the beta-actin expression rate increased after one day of suspension. At the same time, the beta-actin content increased in the membranous fraction of cardiomyocytes and remained at the reference level in the cytoplasm fraction.

Changes in the non-muscle actin content in the fibers of the soleus muscle were similar after 12 and 18 hours of antiorthostatic suspension; however, a decrease of beta- and gamma-actin expression rates was reported much earlier–as soon as after 12 hours of suspension. Apparently, that was the decrease of expression rates that restricted the non-muscle actin content from its full recovery within the membranous fraction after 18 hours of suspension and facilitated very limited growth of fiber stiffness in comparison to its value registered at the 12-hour time point. A further decrease of expression rates resulted in lower concentrations of beta- and gamma-actin in the membranous fraction compared to controls (but their levels in the cytoplasmic fraction did not differ from the control level).

Thus, one of the first events to occur at the protein level was a redistribution of non-muscle actin isoforms between the membranous and cytoplasmic fractions. However, either in cardiomyocytes, or in the fibers of the soleus muscle this processes will be similar; thus, within the mechanosensitivity level this process cannot provide any substantial difference in cell responses to tension or compression.

### Content and Expression Level of Actin-binding Proteins

The following question still remains unclear: what can cause disassembly of the cortical cytoskeleton after 12 hours of antiorthostatic suspension? Disintegration of the cortical cytoskeleton may be initiated by dissociation of proteins from its structure (these proteins bind stress fibrils to each other and fix them to the membrane). Earlier we suggested the hypothesis that dissociation of actin-binding proteins might occur because of deformations of the cortical cytoskeleton due to changes in external mechanical load [53].

As calcium is one of the main secondary messengers, within a large number of actin-binding proteins, we drew our attention exclusively to the calcium-sensitive alpha-actinin isoforms. Alpha-actinin-1 and alpha-actinin-4 are able to bind calcium ions in micromolar concentrations; intracellular calcium concentrations over 10^−7^ M fully inhibit alpha-actinin binding to actin [54], as well as tyrosine phosphorylation with focal adhesion kinase within the actin-binding domain [55].

Very limited data is available about the role of non-muscle alpha-actinin isoforms in skeletal muscles cells and cardiomyocytes. So, it is well established that alpha-actinin-1 is expressed in cardiomyocytes [56], as well as in skeletal muscle cells concomitantly with alpha-actinin-4 at different stages of differentiation [57]. Alpha-actinin 1 and alpha-actinin-4 are non-muscle isoforms of alpha-actinin, a protein of the spectrin family [58]. They function as antiparallel homodimers to bind actin filaments to each other [59]. Alpha-actinin-4 binds the actin cytoskeleton to a membrane and provides interactions between the cortical cytoskeleton and cytoplasmic signaling proteins. At the same time, alpha-actinin-1 is located along stress fibrils, microfilament bundles and focal adhesion zones [60].

We studied alpha-actinin-1 and alpha-actinin-4 content in membranous and cytoplasmic fractions during the whole period of antiorthostatic suspension. In cardiomyocytes, a decrease of alpha-actinin-1 concentration in the membranous fraction (by 49.6%) with its concomitant increase in the cytoplasmic fraction (by 56.4%) were reported after 6 hours of antiorthostatic suspension. At the same time, alpha-actinin-4 concentrations in the membranous and cytoplasmic fractions of cardiomyocytes changed only after 24 hours of suspension. Upon 72 hours of suspension, concentrations of alpha-actinin-1 and alpha-actinin-4, as well as non-muscle actin remained elevated. This finding may be related to formation of the “enhanced” structure of the cortical cytoskeleton in cardiomyocytes of rats during the early stages of suspension. At the same time, alpha-actinin-4 concentrations decreased by 27.4% in the membranous fraction and increased by 28.2% in the cytoplasmic fraction of fibers of the soleus muscle of rats after 6 hours of suspension. The alpha-actinin-1 content changed only after 24 hours of gravity-force load.

Changes in the alpha-actinin-1 content found in cardiomyocytes were more pronounced than changes in the alpha-actinin-4 content found in fibers of the soleus muscle. According to the proposed evaluations [61], such an observation may be linked to a more profound deformation of cardiomyocytes under conditions of antiorthostatic suspension. On the other hand, despite the fact that alpha-actinin-4 possesses a less marked ability to dissociate than alpha-actinin-1, the first changes in expression rates were observed after 6–12 hours in the fibers of the soleus muscle and after 12–18 hours in cardiomyocytes.

It is possible that dissociation of alpha-actinin-1 and alpha-actinin-4 from the cortical cytoskeleton may initiate different signaling pathways. Alpha-actinin-1 and alpha-actinin-4 are able to interact with a large number of different proteins and signaling molecules [62], [63], [64]. But the capability to penetrate directly inside the nucleus and to bind to the promoter region containing the following sequence GCTGCCGCAC-(N4-20)-GGSCGYGGG was demonstrated only for alpha-actinin-4 [57]. Moreover, alpha-actinin-4 interacts with nuclear proteins [65]. Probably, the earlier changes in the transcription profile in fibers of the soleus muscle may be linked with a shorter (or more specific) signaling pathway, mediated by alpha-actinin-4.

## Conclusion

An elevated external mechanical load will naturally result in an increased capability of cells to resist it (as well as in enhancement of cell stiffness and development of the cortical cytoskeleton). Attenuated mechanical load does not require an enhanced cytoskeleton (as a result cell stiffness may be reduced).

Summarizing the obtained results, we can suggest that under increased external mechanical stress alpha-actinin-1 molecules partially dissociate from the cortical cytoskeleton into the cytoplasm, non-muscle actin and alpha-actinin content increase in the membranous fraction and the cell stiffness enhances. When external mechanical stress decreases, alpha-actinin-4 molecules dissociate from the cortical cytoskeleton, non-muscle actin and alpha-actinin content decrease, such as, cell stiffness.
